# Electroacupuncture attenuates intestinal epithelial ferroptosis in inflammatory bowel disease via Piezo1-mediated mitochondrial homeostasis

**DOI:** 10.1186/s13020-025-01218-7

**Published:** 2025-10-06

**Authors:** Haolong He, Jingying Zhou, Sihui Cao, Weiai Liu, Zhigang Mei, Mi Liu

**Affiliations:** 1 Department of Acupuncture-Moxibustion, Tuina and Rehabilitation, The Second Clinical School of Chinese Medicine, Hunan University of Chinese Medicine, Changsha, China; 2 Department of Acupuncture & Moxibustion, School of Acupuncture-Moxibustion, Tuina and Rehabilitation, Hunan University of Chinese Medicine, Changsha, China; 3 Academy of Chinese Medical Sciences, Hunan University of Chinese Medicine, Changsha, China; 4 Key Laboratory of Acupuncture & Moxibustion Bioinformatics, School of Acupuncture-Moxibustion, Tuina and Rehabilitation, Hunan University of Chinese Medicine, Changsha, China

**Keywords:** Electroacupuncture, Inflammatory bowel disease, Piezo1, Ferroptosis, Mitochondrial homeostasis

## Abstract

**Background:**

Inflammatory bowel disease (IBD) involves pathological mechanical forces transduced by mechanosensitive Piezo1 channels. While electroacupuncture (EA) alleviates IBD injury, its relationship with Piezo1-mediated ferroptosis remains unknown.

**Methods:**

Dextran sulfate sodium (DSS)-induced IBD mice and mechanically stressed HIEC-6 intestinal epithelial cells received EA or pharmacological modulators. Pathological scoring, transmission electron microscopy (TEM), inflammatory cytokine assays, Western blotting, and immunofluorescence evaluated mitochondrial dynamics and ferroptosis markers to elucidate the Piezo1-ferroptosis axis and EA's regulatory role.

**Results:**

EA significantly reduced disease activity index (DAI), histopathological scores, colon shortening, and pro-inflammatory cytokines in IBD mice. By inhibiting fission, indicated by a decrease in dynamin-related protein 1 (DRP1), and mitophagy, shown by a reduction in Parkinson protein 2 (PARK2), EA maintained mitochondrial homeostasis. This effect was similar to ferroptosis inhibitor ferrostatin-1 (Fer-1). Moreover, EA lessened RSL3-induced exacerbation of ferroptosis. In vitro, mechanical stress or the Piezo1 agonist Yoda1 induced ferroptosis, which was evident from increased acyl-CoA synthetase Long-chain family member 4 (ACSL4), reactive oxygen species (ROS), malondialdehyde (MDA) and Fe^2^⁺ levels, while decreased glutathione peroxidase 4 (GPX4), ferritin (FTH) and glutathione (GSH) levels. Critically, EA inhibited Piezo1 activation and counteracted Yoda1-aggravated epithelial ferroptosis in vivo.

**Conclusion:**

Piezo1-mediated mitochondrial dyshomeostasis critically drives intestinal epithelial ferroptosis in IBD. EA regulates Piezo1 to maintain mitochondrial homeostasis and suppresses ferroptosis, offering a potential therapeutic strategy for IBD.

**Graphical Abstract:**

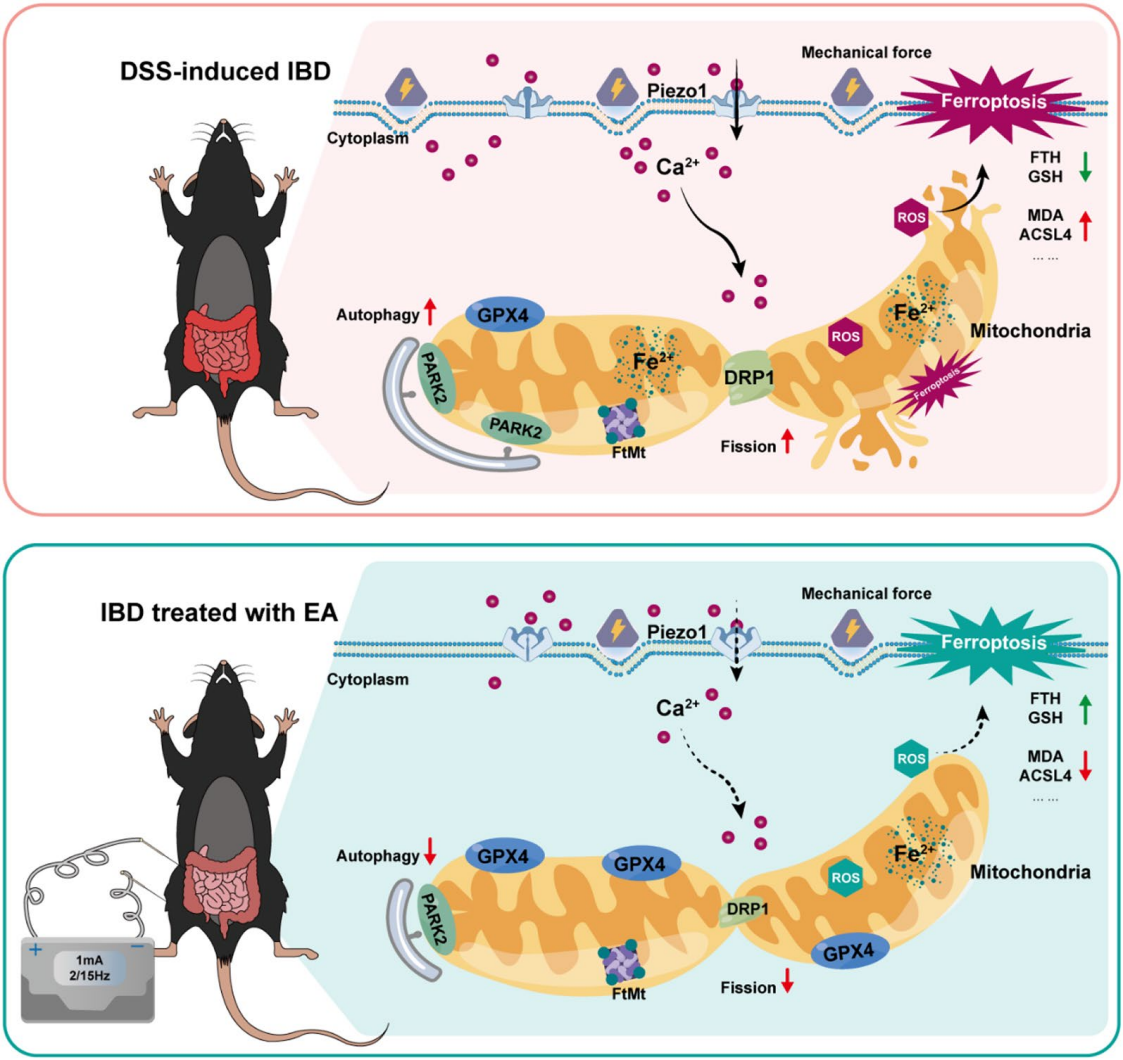

**Supplementary Information:**

The online version contains supplementary material available at 10.1186/s13020-025-01218-7.

## Introduction

Inflammatory bowel disease (IBD), encompassing ulcerative colitis (UC) and Crohn’s disease (CD), represents a group of chronic, nonspecific intestinal inflammatory disorders with incompletely elucidated etiology. Characteristic clinical manifestations include abdominal pain, diarrhea, and mucopurulent bloody stools. As the disease progresses, IBD can lead to debilitating intestinal complications such as fistulas, abscesses, bowel obstruction, and colorectal cancer [1, 2]. Global epidemiological data from 522 population-based studies across 82 regions reveal a persistent rise in IBD incidence over the past three decades, with increasing rates observed across all age groups. The chronic relapsing–remitting course and long-term complications significantly impair patients’ quality of life and work productivity. Often termed a "green cancer" due to its chronic and refractory nature, IBD mortality has gradually increased alongside aging populations, imposing substantial socioeconomic burdens [3, 4]. Current clinical management focuses primarily on inducing and maintaining remission. However, limitations persist, including significant drug-related adverse effects and high postoperative recurrence rates. Notably, electroacupuncture (EA) shows potential benefits in mitigating IBD progression and improving patients' quality of life [5–7]. Bao et al. confirms the efficacy and safety of acupuncture for medication-refractory mild-to-moderate active CD [8]. A clinical trial demonstrated the efficacy of EA in alleviating clinical symptoms in patients with IBD, reducing serum inflammatory factors, and enhancing autonomic activity [5]. A meta-analysis has also demonstrated the efficacy of EA in terms of its curative effect, colonoscopic mucosal lesions, inflammatory factors, and scores related to the Traditional Chinese Medicine syndrome [6].

Nevertheless, the precise pathogenesis of IBD remains incompletely defined. It is widely accepted that dysregulated immune responses, genetic susceptibility, and altered gut microbiota interact intricately to disrupt intestinal mucosal function and drive aberrant immunity [2, 9]. Mechanosensation—the ability to detect mechanical forces and convert them into physiological responses—is essential for normal gastrointestinal function, and its dysregulation is frequently implicated in gastrointestinal pathologies [10]. In IBD patients, pathological alterations such as dysmotility, inflammation, and luminal stenosis generate aberrant biomechanical stimuli within the microenvironment. These forces adversely impact epithelial cells, neurons, and immune cells, contributing to disease progression [11]. Consequently, elucidating IBD pathogenesis and developing strategies to modulate gut mechanotransduction while preserving intestinal barrier integrity represent critical approaches for IBD prevention and treatment.

The gastrointestinal tract is continuously exposed to diverse mechanical stimuli—such as osmotic pressure from luminal contents and peristaltic forces—during digestion and absorption [12]. These biomechanical cues are essential for maintaining normal epithelial function. In IBD patients, common pathological features include intestinal stenosis, mucosal edema, and fibrotic thickening [12, 13]. These alterations create a microenvironment of chronically aberrant mechanical forces, highlighting the critical role of mechanosensation and its dysregulation in IBD pathogenesis. The mechanosensitive cation channel Piezo1 serves as a key mechanotransducer in the gut. In intestinal epithelial cells (IECs), Piezo1 functions as a homeostatic sensor that regulates cellular turnover to preserve epithelial integrity and dynamic equilibrium [14]. Upon activation by mechanical stimuli, Piezo1 transduces these forces into electrochemical signals, exhibiting high permeability to calcium ions (Ca^2^⁺). Piezo1 activation triggers Ca^2^⁺ influx [15], which can induce mitochondrial calcium excess. This calcium overload disrupts mitochondrial function and compromises homeostasis. Mitochondrial impairment leads to reduced antioxidant capacity, characterized by diminished glutathione peroxidase 4 (GPX4) activity and accumulation of lipid peroxides. Concurrently, reactive oxygen species (ROS) promote mitochondrial permeability transition pore (mPTP) opening, amplifying ROS signaling and triggering oxidative stress—a hallmark of ferroptosis [16]. Mounting evidence indicates that ferroptosis contributes to various pathological processes [16, 17]. Crucially, IEC ferroptosis disrupts intestinal barrier function, exacerbating IBD progression. Based on this evidence, we propose that IBD-associated gut mechanosensation initiate a pathological cascade leading to epithelial injury. Aberrant mechanical forces in the IBD microenvironment pathologically overactivate the Piezo1 channel in IECs. This Piezo1 overactivation triggers excessive Ca^2^⁺ influx, resulting in mitochondrial calcium accumulation. The ensuing mitochondrial calcium overload disrupts mitochondrial function and collapses homeostasis, leading to critically reduced antioxidant capacity. This state of severe oxidative stress executes ferroptosis in IECs. The resultant IEC ferroptosis compromises intestinal barrier integrity, ultimately amplifying intestinal inflammation and driving IBD progression.

Substantial evidence confirms that acupuncture modulates physiological balance through multi-level and multi-target mechanisms, correcting immune dysregulation, attenuating inflammatory progression, and thereby promoting colonic mucosal repair and restoring intestinal homeostasis in IBD [8, 18–20]. Notably, EA has been shown to influence the biomechanical microenvironment by regulating intestinal motility, contraction pressure, intraluminal pressure, and enteric neural activity [21–23]. Our prior research utilizing 16S rRNA sequencing and untargeted metabolomics demonstrated that EA effectively improves disease activity index (DAI) and colonic mucosal damage index (CMDI) scores in a rat model of Crohn’s disease (CD). EA significantly reduced colonic and serum inflammatory cytokines while restoring intestinal immune homeostasis through modulation of specific gut microbiota and metabolic profiles, ultimately alleviating mucosal barrier injury [24, 25]. Building on this foundation, the present study establishes ferroptosis as a central driver of IBD pathogenesis and demonstrates that EA protects against intestinal injury by regulating Piezo1-mediated mitochondrial dysfunction and subsequent ferroptosis in IECs. Through a DSS-induced IBD mouse model, we validate that EA antagonizes IEC ferroptosis via mitochondrial homeostasis restoration, mechanistically mediated through the Piezo1 axis, thereby preserving epithelial barrier function through suppression of lipid peroxidation. These results uncover a previously unrecognized mechanotransduction-linked ferroptosis pathway, advancing our understanding of EA’s therapeutic actions in IBD and furnishing pivotal experimental support for refining acupuncture-based clinical interventions.

## Methods and materials

### Animals

Male C57BL/6 J mice (20–22 g, 8 weeks) were obtained from Hunan SJA Laboratory Animal Co., Ltd. (Animal Quality Certificate No. 430727221102888836). Animals were housed in specific-pathogen-free (SPF) facilities at Hunan University of Chinese Medicine (Facility License SYXK[Xiang]2019–0009) maintained at 22 ± 1 °C, 12-h light/dark cycles, with ad libitum access to autoclaved feed and water. All procedures followed institutional guidelines and were approved by the Animal Ethics Committee of Hunan University of Chinese Medicine (Approval No. LL2023060708).

### IBD modeling and validation

**Fig. 1:**
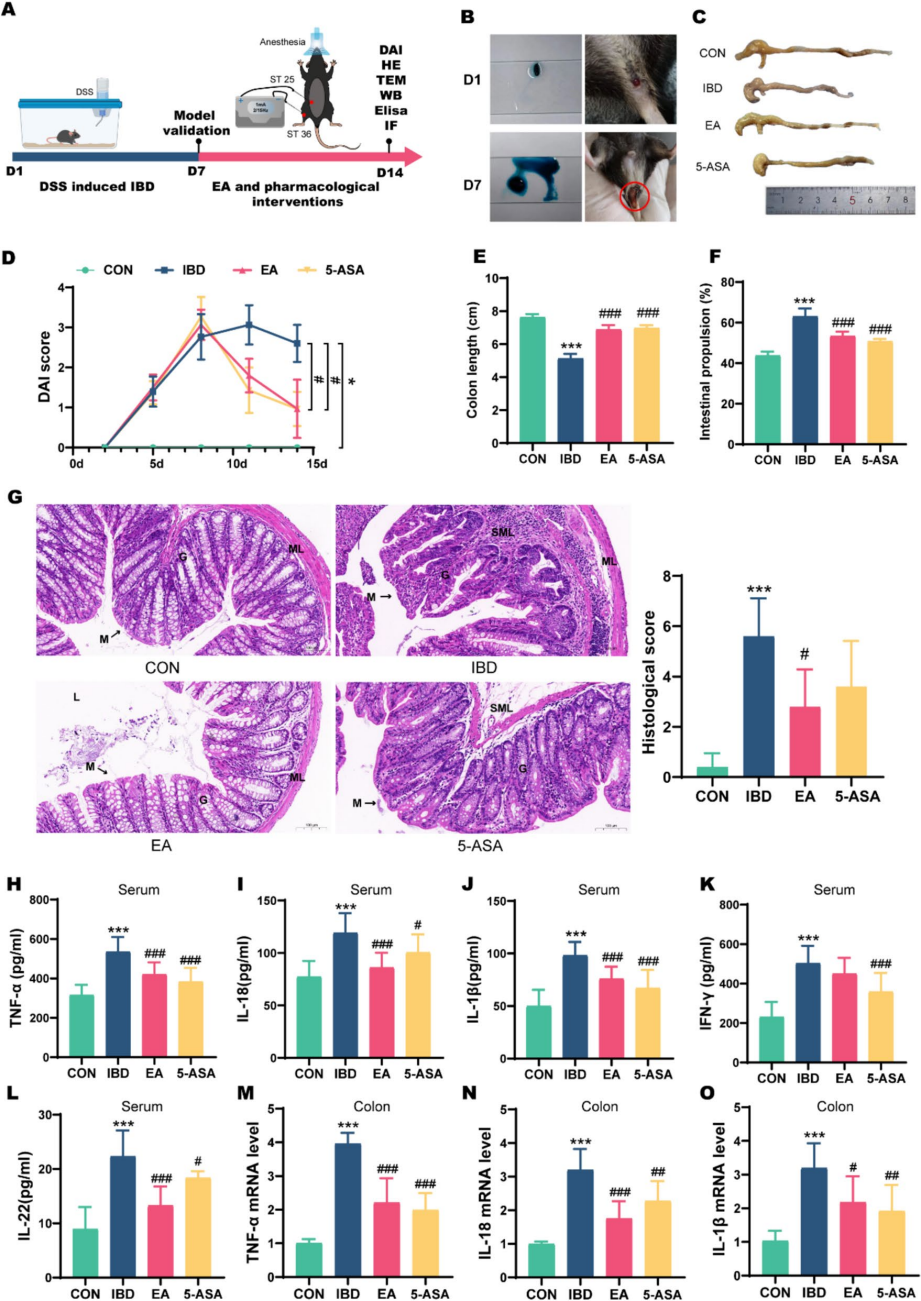
EA attenuates inflammatory pathology in DSS-induced colitis. **A** Schematic illustration of IBD model establishment and EA intervention in mice. **B** Fecal occult blood test in mice with successful IBD model induction. **C** Macroscopic comparison of colon shortening. **D** DAI scores (n = 10). **E** Quantitative analysis of colon length reduction (n = 10). **F** Intestinal propulsion rate (n = 5). **G** HE staining and histopathological scores of colonic tissues. M: mucosa; G: gland; ML: muscular layer; SML: submucous layer; L: lumen. Scale bars: 100 μm (n = 5). **H–L** Serum levels of TNF-α, IL-18, IL-1β, IFN-γ, and IL-22 (n = 10). **M–O** mRNA levels of TNF-α, IL-18, and IL-1β in the colon (n = 8). *p < 0.05, **p < 0.01, ***p < 0.001 vs CON group; # *p* < 0.05, ^##^*p* < 0.01, ^###^*p* < 0.001 vs IBD group

Acute colitis was induced by administering 3% (w/v) dextran sulfate sodium (DSS; MP Biomedicals) in deionized water ad Libitum for 7 days. DSS solutions were prepared fresh every 48 h and protected from Light. After 7 days, two mice per group were randomly sacrificed for colon length measurement and macroscopic/histologic evaluation. IBD modeling was confirmed if mice exhibited worsened diarrhea, mucous stools, fecal occult blood, gross hematochezia, weight loss, and severe colonic congestion, edema, ulceration, and inflammatory infiltration (Fig. 1A, B).

### EA and pharmacological interventions

After modeling was successful and the DAI score was completed, the mice were placed in a 1.5% isoflurane gas mask for inhalation anesthesia, and the warming blanket was adjusted to prevent hypothermia. When vital signs such as breathing and heart rate were stable, the following interventions were performed. In EA protocol, mice were secured in prone position without restraint. Disposable stainless steel filiform needles (0.18 × 13 mm; Suzhou Medical Supplies) were inserted perpendicularly to 3 mm depth at unilateral Zusanli (ST36) and unilateral Tianshu (ST25) acupoints (with alternating use of left and right sides). Paired electrodes from a HANS-200A stimulator (Nanjing Jisheng Medical Technology) were connected to ipsilateral ST36-ST25 points. Stimulation parameters were set at 1 mA constant current, 2/15 Hz dense-disperse wave, inducing mild hindlimb muscle contraction without distress. Treatments lasted 20 min daily for 7 consecutive days (Fig. 1A). For 5-ASA intervention, the mice were administered 5-ASA (#MKCB9194, Sigma) at a dose of 300 mg/kg via intragastric administration, once a day for 7 days. For Fer-1 intervention, 2 h before each intervention, 100 μL of Ferrostatin-1 (Fer-1) (#S724302, Selleck) solution (1 mg/kg) was administered via intraperitoneal injection, continuously for 7 days. For RSL3 intervention, 2 h before each intervention, 100 μL of RSL3 (#S815504, Selleck) solution (5 mg/kg) was administered via intraperitoneal injection, continuously for 7 days. For GsMTx4 intervention, 100 μL of GsMTx4 (#P120502, Selleck) solution (0.5 mg/kg) was administered via intraperitoneal injection every two days, for a total of 7 injections. For Yoda1 intervention, 100 μL of Yoda1 (#S8395, Selleck) solution (0.5 mg/kg) was administered via intraperitoneal injection every two days, for a total of 7 injections.

### General observations and DAI score

Throughout the experimental timeline, daily monitoring included body weight change (%), mental status, activity levels, perianal soiling, and fecal occult blood detection using o-toluidine assay kits (#0824A22, LEAGENE). DAI scores were determined as previously reported, mainly in terms of body mass, fecal characteristics and fecal occult blood in mice [24]. Twenty-four hours after final interventions, five randomly selected mice per group were fasted (water ad libitum) and gavaged with 0.5 mL India ink. Thirty minutes post-gavage, mice were euthanized via cervical dislocation under anesthesia, followed by careful dissection of the entire gastrointestinal tract from stomach to rectum without traction. Intestinal propulsion rate (%) was calculated as (inked length/total intestinal length) × 100.

### HE staining

Colon segments were fixed in 4% paraformaldehyde (PFA) for 24 h, paraffin-embedded, and sectioned at 4 μm thickness. Sections underwent xylene-ethanol gradient deparaffinization, Hematoxylin staining for 5 min, tap water rinsing, eosin counterstaining for 1 min, ethanol dehydration, xylene clearing, and coverslipping. Slides were examined under a light microscope, and pathological damage was scored based on ulceration, inflammatory infiltration, and lesion depth [26].

### Serum cytokine quantification by ELISA

Following interventions, blood samples were collected and allowed to clot at room temperature for 4 h before centrifugation at 12,000 × g for 10 min. Serum aliquots were stored at -80 °C until analysis. Concentrations of TNF-α, IL-1β, IL-18, IL-22, and IFN-γ were determined using commercial ELISA kits (#JL10484, #JL18442, #JL20253, #JL20258, #JL10967, JONLNBIO) according to manufacturers' protocols. Briefly, on the enzyme marker, the OD value of each well is measured after zeroing with a blank control well, a standard curve is made based on the concentration of the standard and the OD value, and then the concentration of the sample is calculated according to the standard curve equation.

### Iron content

The levels of Fe^2^⁺ in colonic tissues were measured using an iron assay kit (#A039-2–1, NJJCBIO). Following the manufacturer's instructions, the absorbance of the standard and sample wells was detected at 562 nm using a microplate reader, and the colorimetric analysis was completed within 1 h.

### Oxidative stress biomarker assays

Malondialdehyde (MDA), reduced glutathione (GSH), and ROS levels in colonic tissues were quantified using commercial kits (#A003-1–1, #A006-2–1, #E004-1–1, NJJCBIO). After following the manufacturer's procedures, the absorbance was measured at 532 nm for MDA, 412 nm for GSH, and 520 nm for ROS using a spectrophotometer.

### Real-time polymerase chain reaction

A small piece of colonic tissue was cut from the liquid nitrogen. Then, total RNA was isolated from the sample using the TRIZOL reagent method in accordance with the manufacturer's instructions. Following the addition of TRIZOL reagent to the sample, centrifugation at 12,000 rpm for 15 min was performed. Subsequently, the supernatant was separated into another clean centrifuge tube. Then, ethanol was added to precipitate the RNA, which was washed using a washing buffer. Finally, the RNA was purified by adding 50 μL of RNase-free water and stored for later use. The RNA concentration and purity were assessed using a UV–Vis spectrophotometer.

cDNA was synthesized according to the reverse transcription kit instructions. Subsequently, the corresponding Real-Time PCR reaction systems were set up. All the detection indexes were performed in accordance with the programmed settings. The data were analyzed using the 2^-△△CT^ method. The primers were provided by Sangon Biotech (Shanghai), and the primer sequences are as follows: TNF-α (F: CCCCCAGTCTGTATCCTTCTA; R: TCGGAAAGCCCATTTGAGT); IL-1β (F: CCTGTGTTTTCCTCCTTGCCT; R: TGCTGCCTAATGTCCCCTTGA); IL-18 (F: GGACACTTTCTTGCTTGCCA; R: TCATGCAGCCTCGGGTATTC); Piezo1 (F: GCATCTTTCTCAGCCACTAC; R: CCAGGGACTTCTCCTCAATC).

### Transmission electron microscopy

To examine the mitochondrial homeostasis in colonic epithelial cells via electron microscopy, samples were taken from multiple sites with bleeding and ulcers in the colon and fixed in fixative at 4 °C for 6 h. Then, samples were postfixated in 1% osmium tetroxide with 0.1 M phosphate buffer (PB) for 4 h at room temperature, followed by three 15-min washes in 0.1 M PB. After dehydration in a graded alcohol series, samples underwent infiltration with acetone and 812 embedding medium, followed by embedding in a 60 °C oven for 48 h. Ultrathin Sects. (60—80 nm) were prepared, double-stained with uranyl acetate and lead citrate for 15 min each, dried overnight at room temperature, and observed under a transmission electron microscope (HT7800, Hitachi).

### Western blot

Following tissue homogenization in RIPA lysis buffer supplemented with protease inhibitors (#G2007, Servicebio) on ice, samples were centrifuged at 12,000 × g for 15 min at 4 °C. Supernatants were collected for total protein quantification via BCA assay (#QB214754, Thermo), with absorbance measured at 562 nm after 30 min incubation at 37 °C. Equal protein loads (20 μg/lane) were resolved on 12% gradient SDS-PAGE gels at 160 V for 45 min. PVDF membranes (#IPVH00010, Millipore) using ice-cold transfer buffer at 350 mA for 60 min. Membranes were blocked in 5% non-fat dry milk/TBST for 1 h at 25 °C with agitation, then incubated overnight at 4 °C with primary antibodies against mitochondrial ferritin (FtMt, # ab66111, Abcam), ferritin (FTH, # 11,682–1-AP, Proteintech), dynamin-related protein 1 (DRP1,#), GPX4 (#ab125066, Abcam), Parkinson protein 2 (PARK2, # 14,060–1-AP, Proteintech), Piezo1 (#15,939–1-AP, Proteintech), acyl-CoA synthetase Long-chain family member 4 (ACSL4, #ab155282, Abcam). After three 10-min TBST washes, membranes were probed with HRP-conjugated secondary antibodies (#SA00001-1, # SA00001-2, Proteintech) for 2 h at room temperature. Following final washes, blots were developed with ECL substrate (#170–5060, Bio-Rad) and imaged using a ChemiDoc MP system (Bio-Rad). Band intensities were quantified in ImageJ.

### Immunofluorescence

Colon segments were fixed in 4% PFA for 24 h, paraffin-embedded, and sectioned at 4 μm thickness. Following xylene-ethanol gradient deparaffinization, antigen retrieval was performed in pH 9.0 EDTA buffer using microwave irradiation (5 min high power to boiling, 15 min low power maintenance), with 30 min cooling to room temperature and 37 °C elution. Sections were encircled with hydrophobic barriers (ImmHis™ Pen, #36310ES64), quenched with 3% H₂O₂ for 10 min, and blocked with 10% species-matched serum for 30 min at room temperature. Primary antibodies were applied overnight at 4 °C in a humidified chamber, followed by three PBST washes and incubation with secondaries for 50 min at room temperature. After additional washes, sections were counterstained with DAPI (#G1012, Servicebio), and imaged using a Digital Slice Scanner (Pannoramic MIDI, 3DHISTECH). Quantitative analysis of mean fluorescence intensity was performed in ImageJ by measuring integrated optical density (IOD) across five random non-overlapping fields per sample.

### Cell culture and treatment

Human intestinal epithelial cells (HIEC-6) were cultured in DMEM (#BL304A, Biosharp) supplemented with 10% FBS (# AB-FBS-0500, ABW) and 1% penicillin–streptomycin at 37 °C/5% CO₂. For revival, cryopreserved cells were thawed in a 37 °C water bath, centrifuged at 300 × g for 5 min, resuspended in complete medium, and plated in 6-cm dishes. At 80% confluence, cells were passaged using 0.25% trypsin–EDTA with neutralization by complete medium, followed by 1:3 subculturing after centrifugation (300 × g, 5 min). Mechanical and pharmacological interventions are as follows: Control (CON) group: Standard culture for 3 h. Mechanical force (MF) group: Cells subjected to 6 kPa cyclic tensile stress at 1 Hz for 1 h using a biomimetic pressure device (NK-P40, Naturethink) after 2 h pre-incubation. MF + GsMTx4 group: Pre-treated with 5 μM GsMTx4 for 2 h, washed, then mechanically stressed as above. Yoda1 group: Stimulated with 5 μM Yoda1 for 2 h followed by standard culture. Fer-1 group: Exposed to 10 μM Fer-1 for 2 h then standard culture. MF + Fer-1 group: Fer-1 pre-treated for 2 h, washed, then subjected to mechanical stress.

### Statistical analysis

All data analyses were performed using SPSS 25.0. Normally distributed data are presented as mean ± standard deviation and analyzed with paired t-tests (two-group comparisons) or one-way ANOVA (multi-group comparisons). For ANOVA with homogeneous variance (verified by Levene's test), post hoc comparisons used LSD or SNK methods; non-homogeneous data employed Tamhane’s T2 or Dunnett’s T3 corrections. Non-normally distributed data are expressed as median (interquartile range) and analyzed using non-parametric Kruskal–Wallis tests. Correlation matrices were generated via Pearson’s coefficient analysis and visualized as Heatmaps using R 4.2.2 (ggplot2 package). Significance was set at *p* < 0.05 for all tests.

## Results

### EA attenuates inflammatory pathology in DSS-induced colitis

Consistent with established protocols, we successfully established an IBD mouse model using 3% DSS followed by EA intervention (Fig. 1A). After 7 days of DSS administration, mice exhibited markedly reduced activity, significant weight loss, perianal mucopurulent discharge, and loose/watery stools. Fecal occult blood tests were strongly positive, accompanied by shortened colon length, elevated DAI scores (*p* < 0.001), and increased intestinal transit rate (*p* < 0.001) (Fig. 1B–F). Histological assessment revealed extensive mucosal damage characterized by epithelial erosion, glandular atrophy, crypt distortion, dense inflammatory infiltrates, and fissuring ulcers. Colonic histological injury scores were significantly increased (*p* < 0.001) (Fig. 1G). Concurrently, serum and colonic levels of pro-inflammatory cytokines (TNF-α, IL-18, IL-1β) were markedly elevated (*p* < 0.001) (Fig. 1H–O). Electroacupuncture intervention substantially mitigated these pathological changes. Compared to the IBD group, EA-treated mice showed improved weight gain, reduced DAI scores, attenuated colon shortening, and normalized intestinal propulsion (*p* < 0.001) (Fig. 1C–F). Histological analysis demonstrated preserved epithelial continuity with minimal ulceration, reduced glandular distortion, and diminished inflammatory cell infiltration, resulting in significantly lower histological injury scores (*p* = 0.02) (Fig. 1G). Notably, EA downregulated TNF-α, IL-18, and IL-1β expression in serum (all *p* < 0.001) and colon tissues (all *p* < 0.05) to levels comparable with 5-ASA treatment efficacy (all *p* < 0.05) (Fig. 1H–O).

### EA suppresses ferroptosis by restoring mitochondrial homeostasis in colonic epithelium

**Fig. 2:**
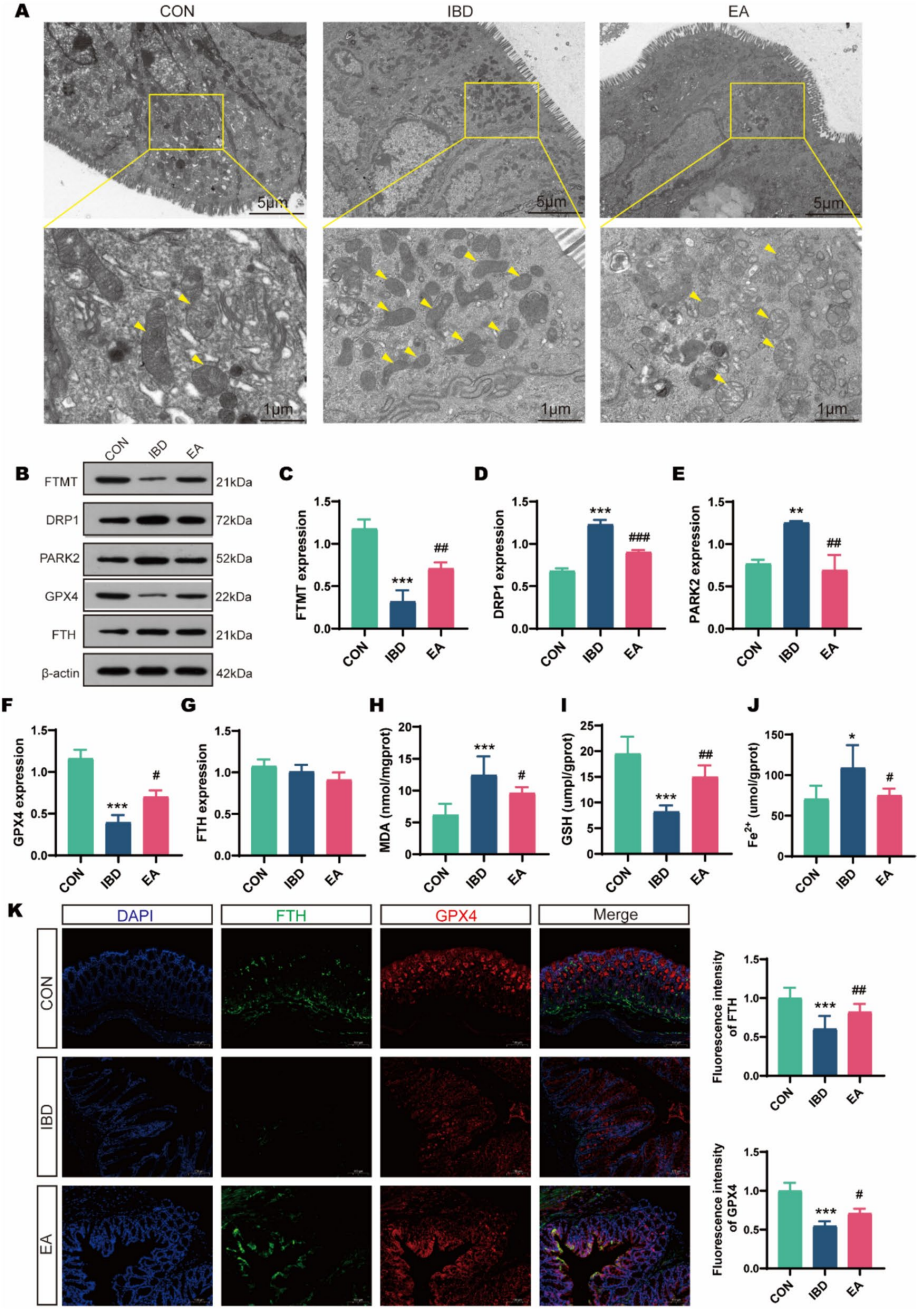
EA suppresses ferroptosis by restoring mitochondrial homeostasis in the colonic epithelium. **A** Transmission electron microscopy (TEM) of mitochondrial ultrastructure in colonic epithelial cells. Yellow arrows indicate ferroptosis-related mitochondria. Scale bars: 5 μm (top), 1 μm (bottom). **B** Western blot bands. **C**–**G** Western blot analysis of FtMt, DRP1, PARK2, GPX4, and FTH expression (n = 3). **H**–**J** Biochemical assays for MDA, GSH, and Fe^2^⁺ levels (n = 5). **K** Immunofluorescence analysis of FTH and GPX4 expression in the colon. Scale bars: 100 μm (n = 5). *p < 0.05, **p < 0.01, ***p < 0.001 vs CON group; ^#^*p* < 0.05, ^##^*p* < 0.01, ^###^*p* < 0.001 vs IBD group

Given that mitochondrial homeostasis imbalance and dysfunction are key triggers of cell ferroptosis, we explored this as a potential target for EA intervention in IBD. Transmission electron microscopy was employed to examine the ultrastructure of mitochondria within the colonic epithelial cells of mice. In the IBD group, significant pathological alterations were observed in the colonic epithelial cells, characterized by moderate cellular edema, localized serrated nuclear invagination, and pronounced mitochondrial damage suggestive of ferroptosis. Notably, some mitochondria exhibited shrinkage, increased electron density of the membrane and matrix, cristae dilation, membrane damage, matrix leakage, and indistinct tight and intermediate junctions, accompanied by numerous autophagic lysosomal structures. Conversely, the enterocytes in the EA group maintained intact cell membranes, displayed mildly swollen mitochondria with preserved membranes, a slightly lighter matrix, shorter cristae, and intact tight, intermediate, and desmosome junctions, with a reduced incidence of ferroptotic mitochondria (Fig. 2A). Subsequent measurements were conducted on several markers intimately associated with mitochondrial homeostasis and ferroptosis. In comparison to the CON group, the IBD group exhibited significantly elevated levels of DRP1 (*p* < 0.001) and PARK2 (*p* = 0.003) protein expression, as well as increased concentrations of MDA (*p* < 0.001) and Fe^2^⁺ (*p* = 0.029), while demonstrating reduced expression of FtMt (*p* < 0.001) and GPX4 (*p* < 0.001), alongside diminished GSH (*p* < 0.001) content in the colon. Conversely, relative to the IBD group, the EA group displayed markedly reduced DRP1 (*p* < 0.001) and PARK2 (*p* = 0.002) protein expression, and lower levels of MDA (*p* = 0.028) and Fe^2^⁺ (*p* = 0.038), coupled with enhanced FtMt (*p* = 0.009) and GPX4 (*p* = 0.013) protein expression, and elevated GSH (*p* = 0.002) content (Fig. 2B–J). The immunofluorescence findings corroborated these observations, indicating a decrease in FTH (*p* < 0.001) and GPX4 (*p* < 0.001) expression in IBD mice, which was subsequently increased following EA intervention (all *p* < 0.05) (Fig. 2K). These findings imply that EA may mitigate ferroptosis in IBD by modulating mitochondrial homeostasis.

### Ferroptosis inhibition as a potential mechanism of EA in IBD treatment

**Fig. 3:**
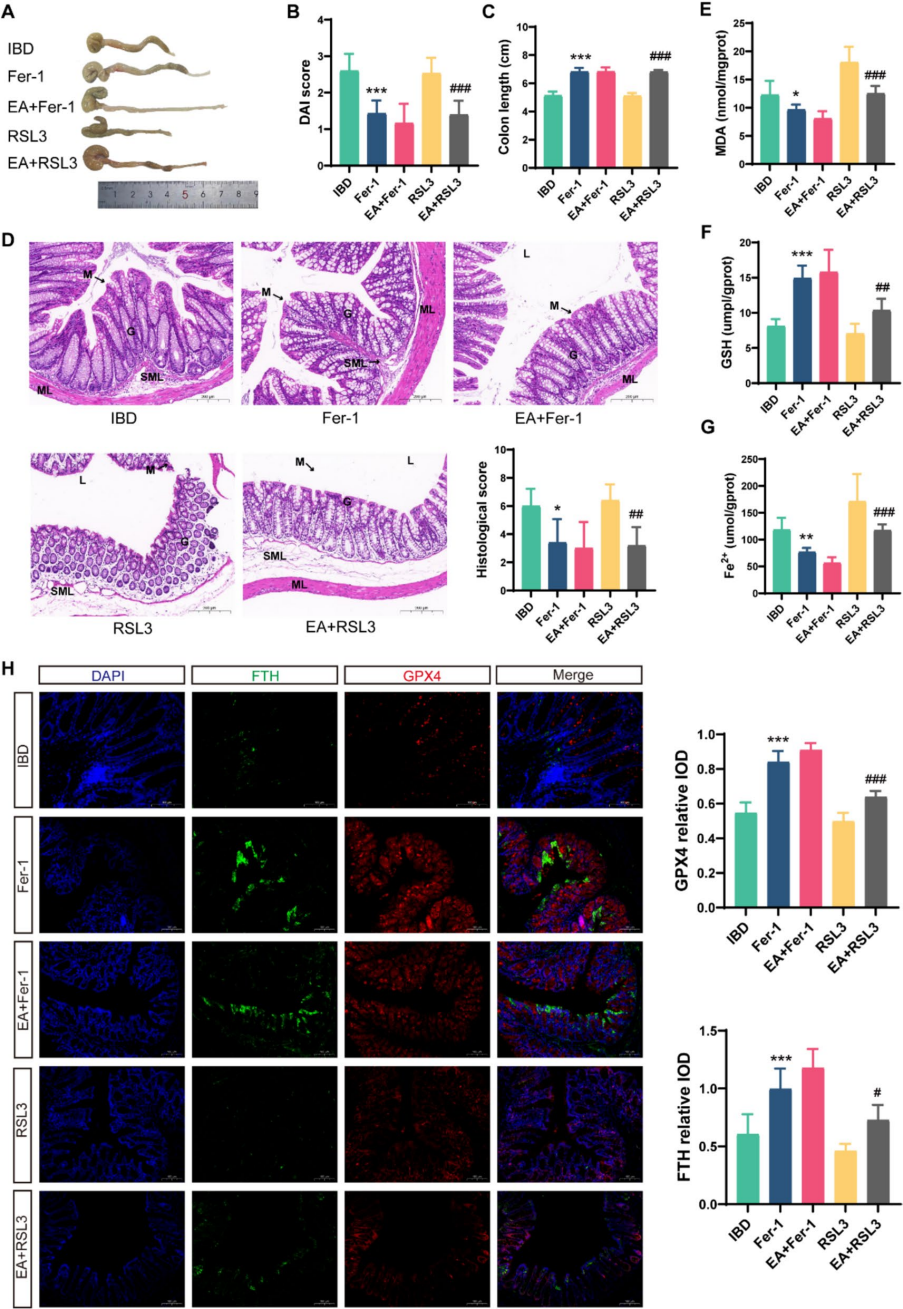
Ferroptosis inhibition as a potential mechanism of EA in IBD treatment.** A** Macroscopic comparison of colon shortening. **B** DAI scores (n = 10). **C** Quantitative analysis of colon length reduction (n = 10). **D** HE staining and histopathological scores of colonic tissues. M: mucosa; G: gland; ML: muscular layer; SML: submucous layer; L: lumen. Scale bars: 100 μm (n = 5). **E–G** Biochemical assays for MDA, GSH, and Fe^2^⁺ levels (n = 5). **H** Immunofluorescence analysis of FTH and GPX4 expression in the colon (n = 5). Scale bars: 100 μm. *p < 0.05, **p < 0.01, ***p < 0.001 vs IBD group; ^#^*p* < 0.05, ^##^*p* < 0.01, ^###^*p* < 0.001 vs RSL3 group

Given that our previous experiments demonstrated EA could alleviate colonic injury in IBD mice, and considering the strong association between IBD and ferroptosis, we delved deeper using ferroptosis inhibitor Ferrostatin-1 (Fer-1) and inducer RSL3 to clarify EA's role in regulating ferroptosis in IBD mice. First, Fer-1 intervention significantly reduced DAI scores (*p* < 0.001), ameliorated colon shortening (*p* < 0.001), and alleviated pathological damage (*p* = 0.033) (Fig. 3A–D). Biochemically, Fer-1 decreased MDA (*p* = 0.024) and Fe^2^⁺ accumulation (*p* = 0.009) while elevating GSH levels (*p* < 0.001) (Fig. 3E–G). Immunofluorescence confirmed upregulated FTH (*p* = 0.001) and GPX4 (*p* < 0.001) expression in Fer-1-treated colons (Fig. 3H), demonstrating ferroptosis inhibition analogous to EA's effects. We then examined whether EA could further enhance Fer-1's protective effects. Interestingly, both gross colon observation and HE staining showed that EA failed to further ameliorate pathological damage in Fer-1-treated IBD mice. Macroscopic and histological evaluations revealed comparable colon shortening, ulceration, and injury scores between Fer-1 and EA + Fer-1 groups (all *p* > 0.05) (Fig. 3A–D). Similarly, on the basis of inhibiting colonic ferroptosis by Fer-1, EA could not further reduce MDA (*p* = 0.296) and Fe^2^⁺ (*p* = 0.337) levels or increase GSH content (*p* = 0.743) (Fig. 3E–G). Consistently, immunofluorescence detected no incremental increase in FTH or GPX4 signal intensity in the EA + Fer-1 group versus Fer-1 alone (all *p* > 0.05) (Fig. 3H). To further validate the role of ferroptosis in EA's mechanism, we administered RSL3 to exacerbate ferroptosis. RSL3 administration significantly aggravated colonic pathology in IBD mice, manifesting as severe colon shortening, congestive edema, epithelial denudation, deep penetrating ulcers, and loss of goblet cells and crypts. Their DAI and histological scores were elevated (Fig. 3A–D). Crucially, subsequent EA intervention attenuated these changes, reducing MDA (*p* < 0.001) and Fe^2^⁺ accumulation (*p* < 0.001) while restoring GSH levels (*p* = 0.005) (Fig. 3E–G). Immunofluorescence analysis further demonstrated that, compared to the RSL group, FTH (*p* = 0.028) and GPX4 (*p* < 0.001) expression improved in the EA + RSL group, indicating EA could counteract RSL3's pro-ferroptotic effects (Fig. 3H). Collectively, these results strongly suggest that EA alleviates IBD colonic epithelial injury by inhibiting cellular ferroptosis.

### Piezo1 mediates IBD pathogenesis and is attenuated by targeted inhibition

**Fig. 4:**
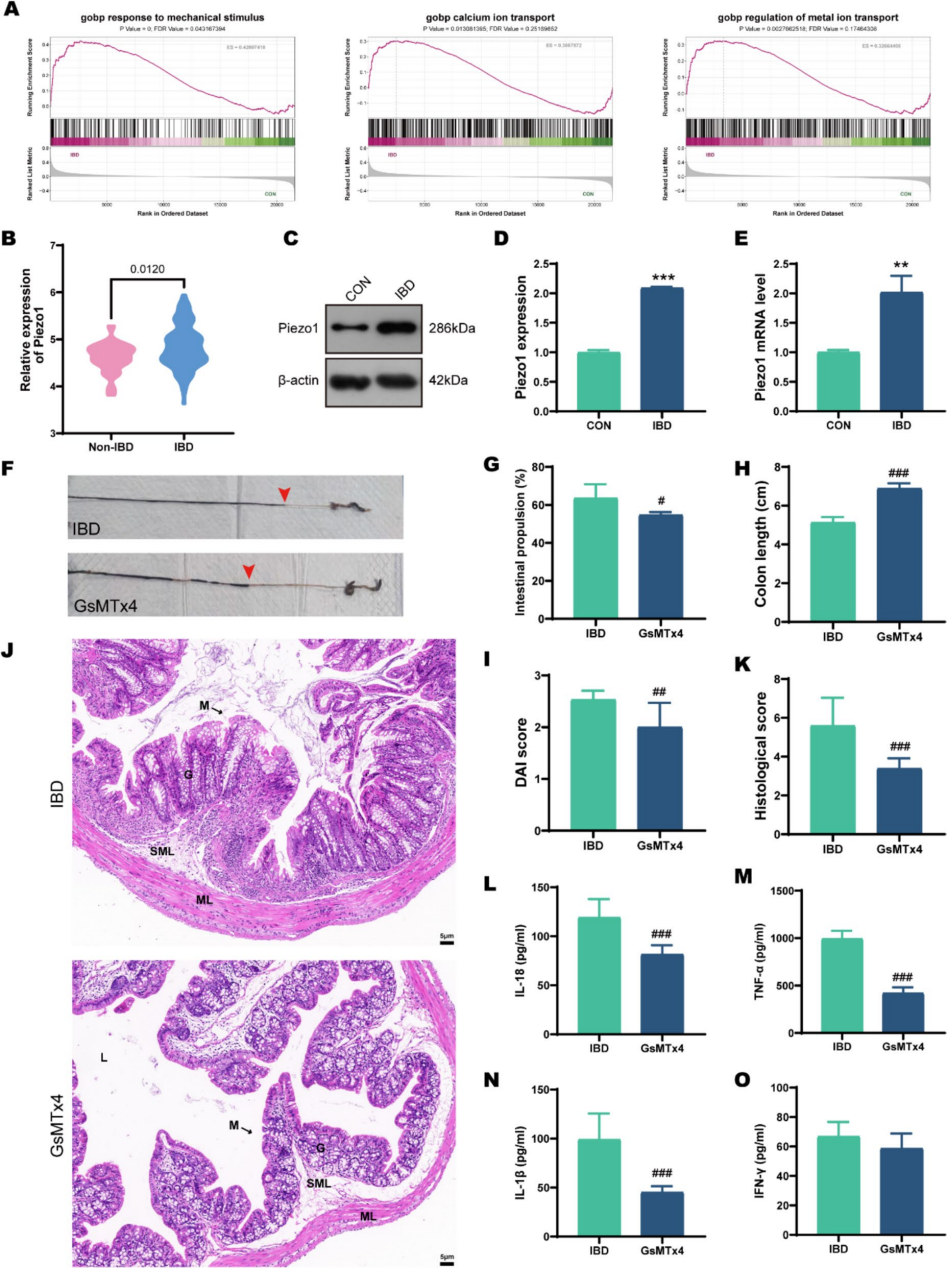
Inhibiting Piezo1 alleviates IBD. **A** GSEA plot illustrating the enrichment of gene sets in IBD patients from dataset GSE112366. **B** Piezo1 expression in the ileum of IBD and non-IBD patients from dataset GSE112366. **C**–**E** Western blot and qRT-PCR analysis of Piezo1 protein and mRNA expression in IBD mice treated with GsMTx4 (n = 3). **F** Macroscopic colon observation and ink propulsion assay. Red arrows indicate the position of ink propulsion. **G** Intestinal propulsion rate (n = 3). **H** Colon length comparison (n = 10). **I** DAI scores (n = 10). **J-K** HE staining and histopathological scores of colonic tissues. M: mucosa; G: gland; ML: muscular layer; SML: submucous layer; L: lumen. Scale bars: 5 μm (n = 5). **L-O** The levels of the inflammatory factors IL-18, TNF-α, IL-1β, and IFN-γ were examined (n = 5). *p < 0.05, **p < 0.01, ***p < 0.001 vs CON group; ^#^*p* < 0.05, ^##^*p* < 0.01, ^###^*p* < 0.001 vs IBD group

Previous research has indicated that Piezo1 expression is elevated in the ileum of IBD patients compared to healthy individuals [27], suggesting Piezo1 may be a novel therapeutic target for IBD. We obtained the IBD-related dataset GSE112366 from the GEO database and analyzed the gene expression profiles of ileal tissue samples from 141 moderately-to-severely ill IBD patients and 26 non-IBD subjects. Gene Set Enrichment Analysis (GSEA) revealed that gene sets related to Piezo1, such as "response to mechanical stimulus," "calcium ion transport," and "regulation of metal ion transport," were enriched in IBD patients (Fig. 4A). Additionally, Piezo1 expression was significantly higher in IBD patients than in non-IBD subjects (Fig. 4B). We then validated this in DSS-induced IBD mice. Western blotting and RT-qPCR confirmed upregulated Piezo1 protein (*p* < 0.001) and mRNA (*p* = 0.003) levels in colonic tissues compared to the CON group, which is consistent with findings from human samples. (Fig. 4C–E). To verify the role of Piezo1 in IBD, we intraperitoneally injected IBD mice with GsMTx4, a selective Piezo1 channel blocker. The results showed that from the perspective of IBD's inflammatory damage, after GsMTx4 intervention, overall indicators tended to improve. For instance, DAI scores decreased (*p* = 0.003), intestinal propulsion rates decreased (*p* = 0.014), pathological damage was alleviated (*p* < 0.001), and concentrations of inflammatory factors, such as IL-18, decreased (*p* < 0.001). These results suggested that blocking Piezo1 can partially alleviate IBD symptoms.

**Fig. 5:**
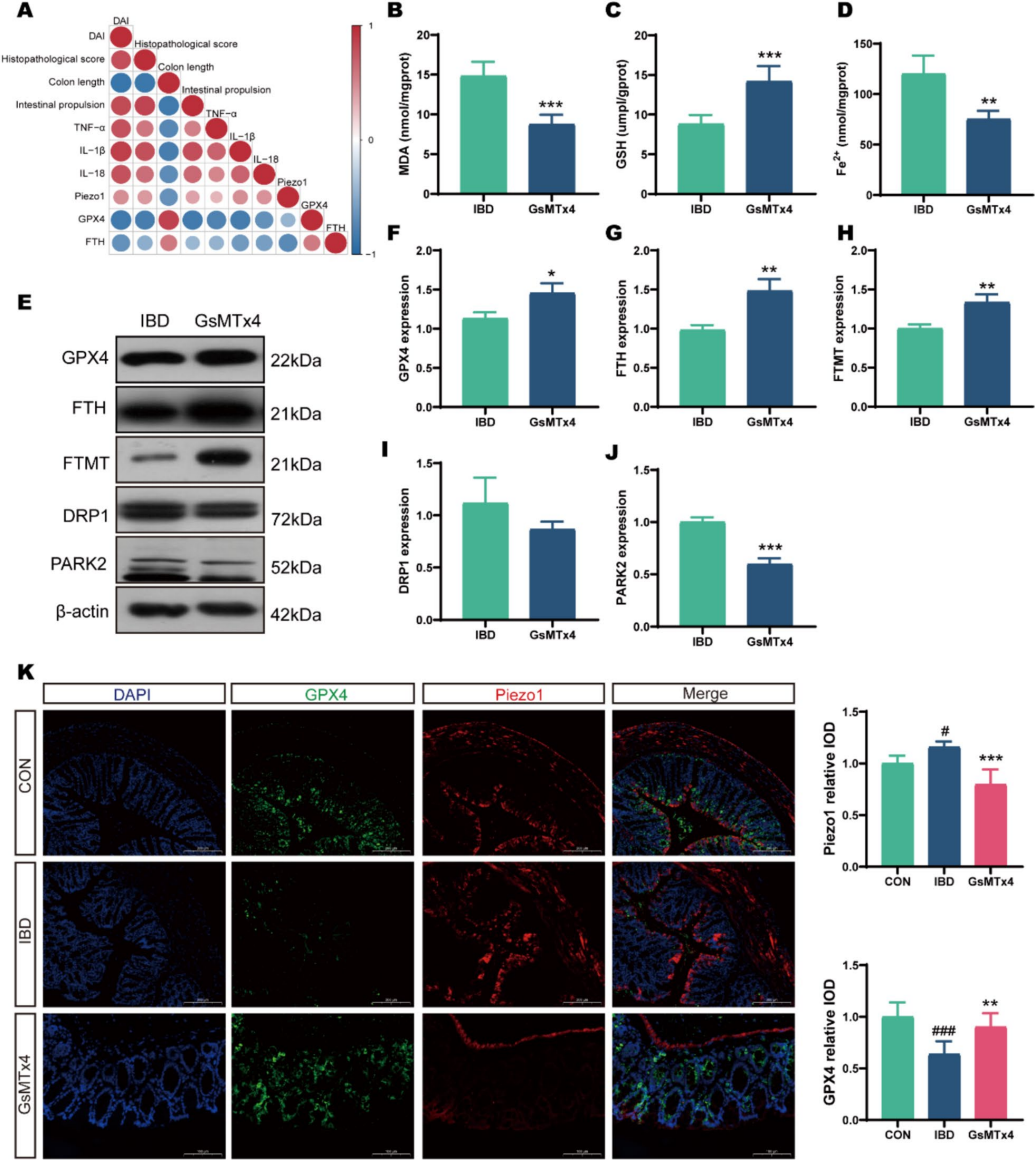
Inhibiting Piezo1 antagonizes ferroptosis in IBD. **A** Correlation heatmap of Piezo1 expression with IBD severity indices and ferroptosis markers. Red indicates a positive correlation and blue a negative correlation, with deeper colors representing stronger correlations. **B**–**D** Biochemical assays for MDA, GSH, and Fe^2^⁺ levels (n = 5). **E** Western blot bands. **F**–**J** Western blot analysis of GPX4, FTH, FtMt, DRP1, and PARK2 expression (n = 3). **K** Immunofluorescence analysis of Piezo1 and GPX4 expression in the colon. Scale bars: 200 μm (n = 5). *p < 0.05, **p < 0.01, ***p < 0.001 vs IBD group; ^#^*p* < 0.05, ^##^*p* < 0.01, ^###^*p* < 0.001 vs CON group

Since our prior experiments established ferroptosis as a critical process in IBD-induced intestinal epithelial injury and demonstrated that EA could mitigate colonic damage by maintaining mitochondrial homeostasis and inhibiting ferroptosis, we propose that Piezo1 may be a potential target in ferroptosis regulation. To explore this, we analyzed the correlations among Piezo1, IBD-related indicators, and key ferroptosis proteins. Correlation analysis revealed strong positive associations between Piezo1 expression and IBD severity metrics, while demonstrating inverse correlations with ferroptosis suppressors GPX4 and FTH (Fig. 5A). Pharmacological inhibition of Piezo1 using GsMTx4 significantly attenuated ferroptotic alterations, reducing MDA (*p* < 0.001) and Fe^2^⁺ (*p* = 0.001) accumulation while elevating GSH (*p* < 0.001) reserves compared to untreated IBD mice (Fig. 5B–D). Regarding key mitochondrial and ferroptosis—related proteins, GsMTx4 treatment significantly elevated expression of ferroptosis regulators GPX4 (*p* = 0.02), FTH (*p* = 0.006), and FtMt (*p* = 0.007), concurrently reducing PARK2 (*p* < 0.001) expression. Although DRP1 downregulation did not reach statistical significance (*p* = 0.16), it consistently trended downward (Fig. 5E–J). These findings indicate that inhibiting Piezo1 may reduce mitochondrial fission and autophagy, stabilize mitochondria, and suppress ferroptosis. Immunofluorescence analysis revealed increased Piezo1 expression in the colon of IBD mice (*p* = 0.013), primarily in the intestinal epithelium and muscularis. GsMTx4 treatment not only diminished Piezo1 signal intensity (*p* < 0.001) but also reciprocally enhanced GPX4 expression (*p* = 0.002) (Fig. 5K). Collectively, these results imply that Piezo1 may drive mitochondrial homeostasis disruption, leading to ferroptosis and exacerbating IBD-related intestinal epithelial injury.

### Mechanical force triggers ferroptosis in intestinal epithelial cells via Piezo1

**Fig. 6:**
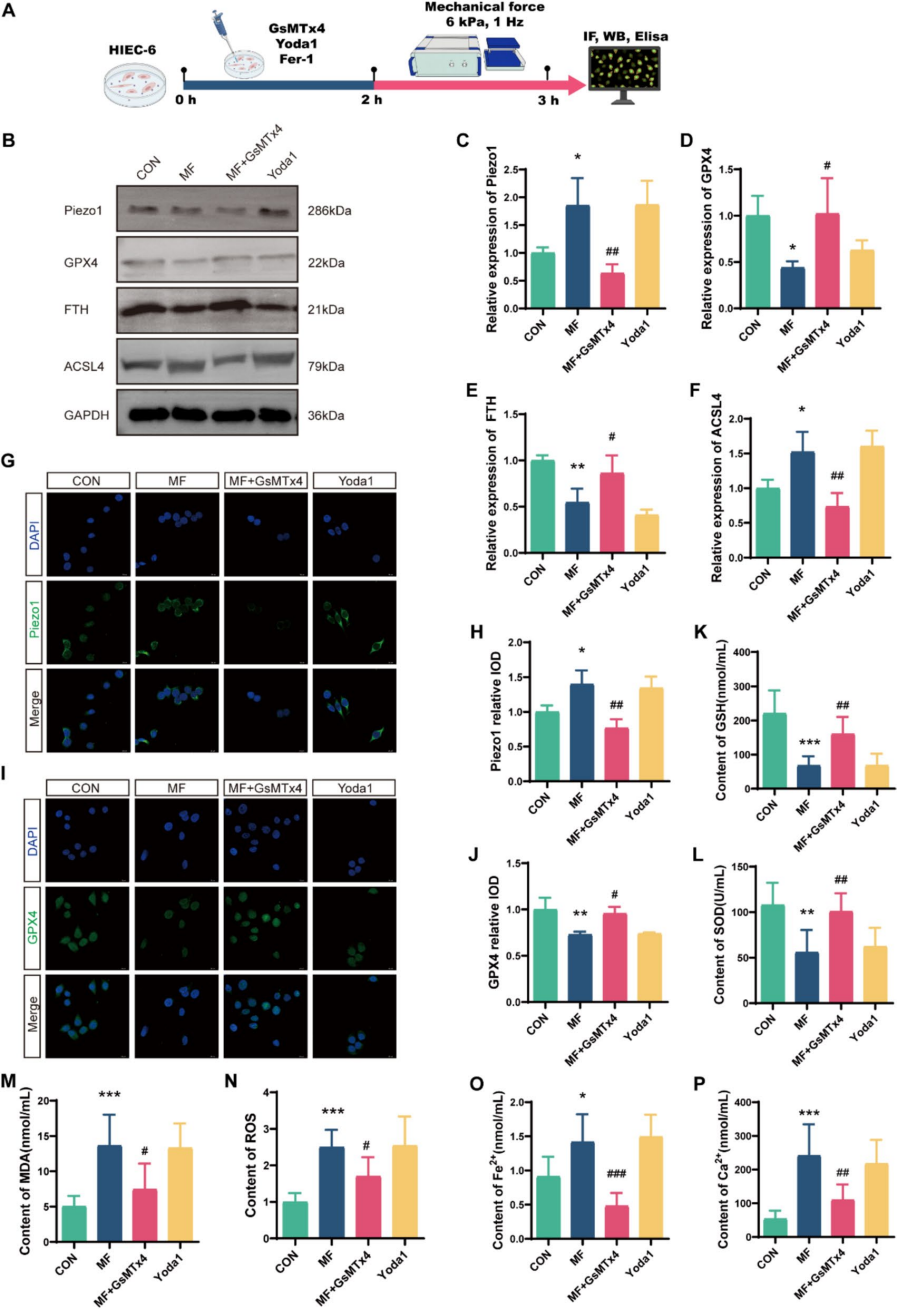
Mechanical force triggers ferroptosis in intestinal epithelial cells via Piezo1. HIEC-6 cells were subjected to mechanical force stimulation or treated with GsMTx4 and Yoda1. **A** The experimental protocol in vitro. **B** Western blot bands. **C-F** Western blot analysis of Piezo1, GPX4, FTH, and ACSL4 expression (n = 3). **G-J** Immunofluorescence analysis of Piezo1 and GPX4 expression in HIEC-6 cells. Scale bars: 25 μm (n = 3). **K–P** Relative levels of GSH, SOD, MDA, ROS, Fe^2^⁺ and Ca^2^⁺ in HIEC-6 cells (n = 6). *p < 0.05, **p < 0.01, ***p < 0.001 vs CON group; ^#^*p* < 0.05, ^##^*p* < 0.01, ^###^*p* < 0.001 vs MF group

**Fig. 7:**
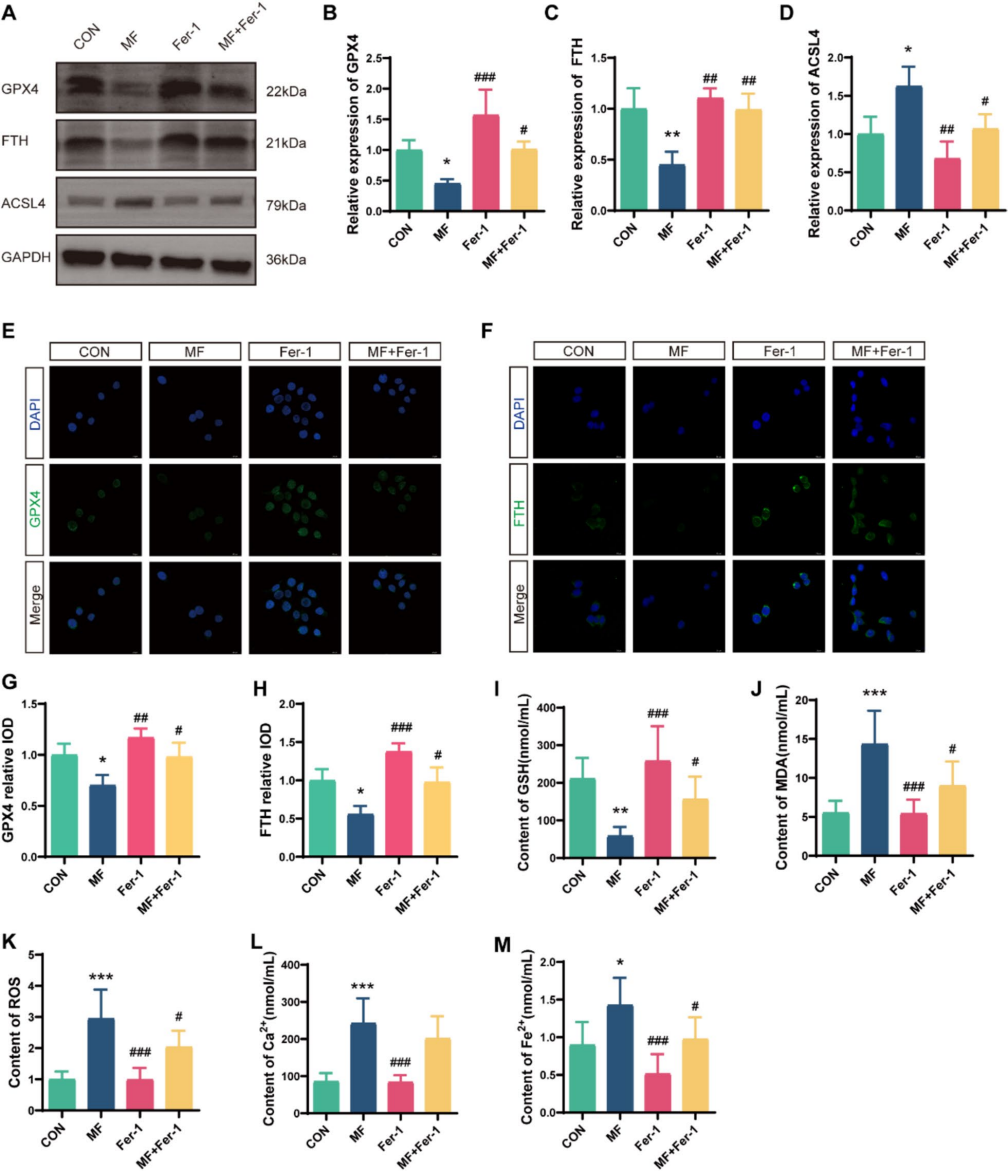
Fer-1 mitigates mechanical force-induced ferroptosis. HIEC-6 cells were subjected to mechanical force stimulation after pretreatment with Fer-1.** A** Western blot bands. **B-D** Western blot analysis of GPX4, FTH, and ACSL4 expression (n = 3). **E–H** Immunofluorescence analysis of GPX4 and FTH expression in HIEC-6 cells. Scale bars: 25 μm (n = 3). **I-M** Relative levels of GSH, MDA, ROS, Ca^2^⁺ and Fe^2^⁺ in HIEC-6 cells (n = 6). *p < 0.05, **p < 0.01, ***p < 0.001 vs CON group; ^#^*p* < 0.05, ^##^*p* < 0.01, ^###^*p* < 0.001 vs MF group

Given that IBD patients frequently experience mechanical force alterations in the gut, such as rapid transit of luminal contents, smooth muscle swelling, and abnormal peristalsis [28], we investigated whether mechanical overstimulation promotes ferroptosis in IECs through Piezo1. In experiments, HIEC-6 cells were subjected to mechanical force using a biomimetic pressure device and treated with the Piezo1 inhibitor GsMTx4 or the agonist Yoda1. Mechanical force significantly upregulated Piezo1 (all *p* < 0.05) and ACSL4 (*p* = 0.044) expression while suppressing GPX4 (all *p* < 0.05) and FTH (*p* = 0.007) levels, as confirmed by western blotting and immunofluorescence (Fig. 6B–J). Concurrently, mechanical loading reduced GSH (*p* < 0.001) and SOD (*p* = 0.002) activity while elevating MDA (*p* < 0.001), ROS (*p* < 0.001), Fe^2^⁺ (*p* = 0.028), and Ca^2^⁺ (*p* < 0.001) accumulation (Fig. 6K–P). Critically, GsMTx4 co-treatment abolished these pro-ferroptotic changes (all *p* < 0.05). Notably, Yoda1 mimicked mechanical force effects, similarly inducing ferroptotic signatures and confirming that Piezo1 activation induces ferroptosis. Subsequently, we cultured mechanically stimulated HIEC-6 cells in the presence or absence of the ferroptosis inhibitor Fer-1. Western blot and immunofluorescence results indicated that Fer-1 treatment reduced ACSL4 expression and increased GPX4 and FTH expression (all *p* < 0.05) (Fig. 7A–H). It also elevated GSH levels and reduced MDA, ROS, Fe^2^⁺, and Ca^2^⁺ levels (all *p* < 0.05) (Fig. 7J–O). These findings confirmed that mechanical force induce ferroptosis in IECs via Piezo1, positioning Piezo1 inhibition as a potential therapeutic strategy for modulating ferroptosis in IBD.

### EA attenuates ferroptosis through Piezo1 regulation in IBD

**Fig. 8:**
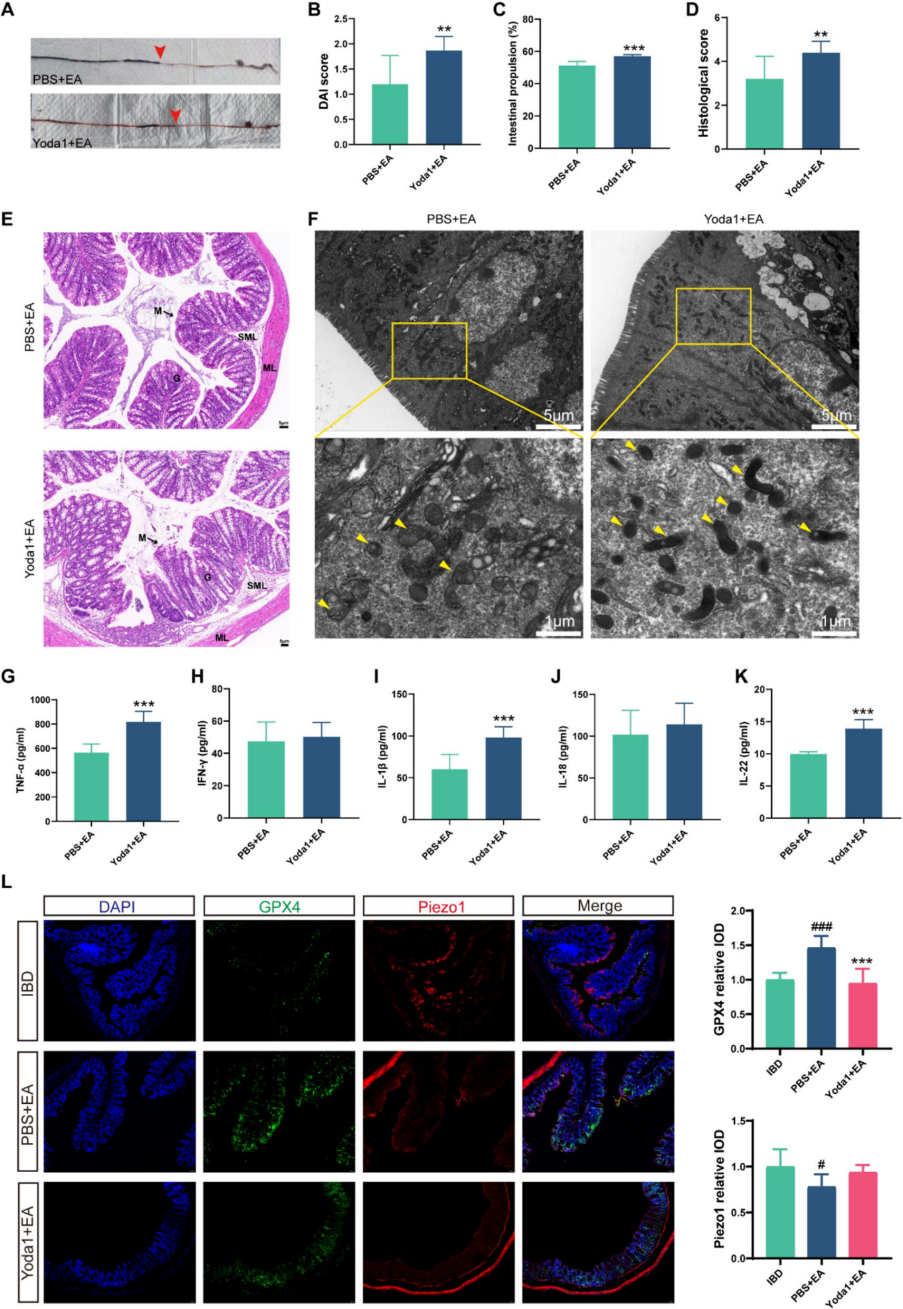
EA attenuates ferroptosis through Piezo1 regulation in IBD. EA was applied to IBD mice treated with Yoda1 or PBS. **A** Macroscopic colon observation and ink propulsion assay. Red arrows indicate the position of ink propulsion. **B** DAI scores (n = 10).** C** Intestinal propulsion rate (n = 3). **D**–**E** HE staining and histopathological scores of colonic tissues. M: mucosa; G: gland; ML: muscular layer; SML: submucous layer; L: lumen. Scale bars: 5 μm (n = 5). **F** TEM of mitochondrial ultrastructure in colonic epithelial cells. Yellow arrows indicate ferroptosis-related mitochondria. Scale bars: 5 μm (top), 1 μm (bottom). **G**–**K** The levels of the inflammatory factors IL-18, TNF-α, IL-1β, and IFN-γ were examined (n = 5). **L** Immunofluorescence analysis of Piezo1 and GPX4 expression in the colon (n = 5). Scale bars: 50 μm. *p < 0.05, **p < 0.01, ***p < 0.001 vs PBS + EA group; # *p* < 0.05, ^##^*p* < 0.01, ^###^*p* < 0.001 vs IBD group

Our research indicates that EA mitigates ferroptosis in intestinal epithelial cells of IBD mice, and Piezo1 inhibition has a similar effect. To explore whether EA exerts its therapeutic effects by targeting Piezo1 in IBD mice, we administered Yoda1 via intraperitoneal injection. Results showed that Yoda1 counteracted EA's therapeutic effects, causing increases in DAI scores (*p* = 0.004), intestinal propulsion rates (*p* < 0.001), and histopathological injury scores (*p* = 0.004) (Fig. 8A–D). HE staining revealed that while EA restored epithelial continuity and reduced inflammatory infiltration, Yoda1 administration disrupted mucosal integrity and intensified inflammatory damage (Fig. 8E). Ultrastructural examination via electron microscopy further demonstrated increased mitochondrial ferroptosis in Yoda1-treated IBD mice (Fig. 8F). Furthermore, the levels of TNF-α, IL-1β, and IL-22 rebounded following Yoda1 intervention, counteracting EA's anti-inflammatory effects (all *p* < 0.001) (Fig. 8G–K). Immunofluorescence confirmed EA-mediated Piezo1 downregulation coupled with GPX4 upregulation in colonic epithelium. Yoda1 treatment not only suppressed GPX4 (*p* < 0.001) expression but also induced a non-significant upward trend in Piezo1 levels (*p* = 0.127) (Fig. 8L). In terms of mitochondrial proteins, Yoda1 decreased FtMt (*p* = 0.047) expression alongside elevated DRP1 (*p* = 0.014) and PARK2 (*p* = 0.043), indicating promoted mitochondrial fission and mitophagy, and disruption of mitochondrial homeostasis (Supplementary Fig. A-D). Yoda1 concurrently antagonized EA's antioxidant capacity by dysregulating MDA (*p* = 0.12), GSH (*p* = 0.017), and Fe^2^⁺ (*p* = 0.002) homeostasis (Supplementary Fig. E–G). These data established that Piezo1 activation functionally counteracts the benefits of EA, confirming that EA targets the Piezo1-mitochondria-ferroptosis axis to protect against epithelial injury in IBD.

## Discussion

This study explores the effect of EA in mitigating ferroptosis in the intestinal epithelium of mice with DSS-induced IBD. By combining pharmacological activation and inhibition with in vitro and in vivo experiments, we reveal that Piezo1 plays a pivotal role in mediating EA's protective effect against IBD pathology. Crucially, we identify mitochondrial homeostasis and ferroptosis as the core downstream pathways through which Piezo1 drives IBD pathogenesis. Our findings establish that EA attenuates intestinal injury by modulating Piezo1-dependent mitochondrial stabilization, thereby inhibiting epithelial ferroptosis.

Ferroptosis, a regulated cell death modality distinct from apoptosis, autophagy, and necrosis, is characterized by membrane integrity collapse, shrunken mitochondria with increased membrane density, uncontrolled lipid peroxidation, GSH depletion, and iron accumulation [29]. Emerging evidence highlights the role of cell death mechanisms in IBD-related intestinal epithelial homeostasis, with ferroptosis emerging as a potential contributor to IBD exacerbation. Research on DSS-induced colitis shows that iron overload worsens intestinal inflammation [30], while iron chelators reduce ROS and improve colon symptoms in IBD mice [31]. In CD patients, impaired GPX4 activity and lipid peroxidation in IECs underscore GPX4's importance in maintaining gut homeostasis [32]. Clinical and animal studies indicate abnormal expression of ferroptosis-related genes/proteins in IBD [33, 34]. Ferroptosis inhibitors like Fer-1, Lip-1, and DFO improve DAI scores and colon length in DSS-induced colitis mice, suggesting ferroptosis involvement in epithelial injury and the potential of its inhibition as a therapeutic strategy [33, 35]. Mechanistically, GPX4 inactivation during ferroptosis leads to lipid peroxidation product accumulation, inducing cell death. These products include lipid hydroperoxides (L-OOHs) and reactive aldehydes like MDA [36]. GPX4 inactivation is also linked to intracellular GSH depletion, as GSH is a GPX4 substrate [37]. Iron accumulation is another hallmark of ferroptosis, with elevated Fe^2^⁺ specifically increasing oxidative stress. Iron regulatory proteins like FTH and FTL form structures that store Fe^3^⁺, maintaining cellular iron balance [38].

Previous research on EA's regulation of ferroptosis has mainly focused on cardiovascular and neurological diseases [39–41]. In this study, using electron microscopy, Western blot, and immunofluorescence, we observed severe mitochondrial damage in colonic epithelial cells of IBD mice, indicative of ferroptosis. This was accompanied by increased MDA and Fe^2^⁺ levels, and decreased GSH, GPX4, and FTH levels. EA or Fer-1 treatment reversed these changes, suggesting they inhibit ferroptosis. Conversely, the ferroptosis inducer RSL3 exacerbated IBD-related ferroptosis, an effect mitigated by EA. Thus, our experiments confirm that EA can suppress ferroptosis in the intestinal epithelial cells of IBD mice.

Under physiological conditions, mitochondrial homeostasis—a dynamic equilibrium of morphology, structure, quantity, volume, quality, and function—is essential for cellular and tissue integrity [42, 43]. However, factors such as peroxide accumulation, calcium overload, and excitatory amino acid toxicity can impair mitochondrial oxidative phosphorylation, increasing ROS production and reducing their clearance, thereby inducing oxidative stress. This imbalance initiates oxidative stress, triggering ROS amplification that damages mitochondrial architecture and collapses homeostasis [44]. As central regulators of oxidative metabolism and cell death, mitochondria are intrinsically linked to ferroptosis execution. Mounting evidence confirms that mitochondrial dyshomeostasis is a prerequisite for ferroptotic initiation [45, 46]. For instance, Wang et al. reported that the mitochondrial-targeted antioxidant Mito- TEMPO blocked doxorubicin-induced cardiac ferroptosis in mice, offering strong in vivo evidence of the link between mitochondria and ferroptosis [46]. Additionally, several mitochondrial antioxidant enzymes, including GPX4, SOD2/MnSOD, and MGST1, play key roles in suppressing ferroptosis [47]. Mitochondrial CoQ_10_ can also effectively prevent ferroptosis. Furthermore, a 2021 Nature study from the University of Texas MD Anderson Cancer Center revealed that mitochondrial GPX4 and DHODH, located on the inner mitochondrial membrane, together form a major ferroptosis defense system. DHODH inhibits ferroptosis by generating CoQH_2_ [48]. Lastly, mitochondrial oxidative damage can release certain mitochondrial apoptosis-regulatory factors, such as AIFM1, which are related to the molecular link between apoptosis and ferroptosis [49].

Dysregulated mitochondrial dynamics and functional impairment heighten cellular susceptibility to ferroptosis, yet mitochondria possess unique defense mechanisms against ferroptosis-induced oxidative damage [50]. As highly dynamic organelles, mitochondria undergo continuous fusion, fission, and mitophagy. Drp1-mediated mitochondrial fission, particularly when activated by ROS, increases ferroptosis vulnerability in vitro [51]. Another key aspect of mitochondrial dynamics is mitophagy, particularly the PINK1 (PARK2)-mediated degradation pathway. Current research indicates that autophagy and mitophagy contribute to ferroptosis [52]. Beyond structural dynamics, mitochondrial iron regulation critically influences ferroptosis through mtDNA release and TCA cycle modulation. Analogous to cytosolic FTH, FtMt forms iron-sequestering spherical structures but uniquely localizes to mitochondria to buffer intra-mitochondrial iron pools [53]. This multifaceted regulatory network underscores that mitochondrial integrity governs cellular antioxidant capacity and ferroptosis resistance. Maintaining mitochondrial homeostasis and function is thus vital for balancing the cellular antioxidant system, counteracting ferroptosis, restoring intestinal epithelial homeostasis, and improving IBD. In our study, electron microscopy of colonic epithelial cells in IBD mice revealed smaller mitochondria with increased membrane and matrix electron density, cristae dilation, membrane damage, matrix leakage, and numerous autophagic lysosomes, all indicative of ferroptosis. Elevated DRP1/PARK2 and diminished FtMt expression indicated excessive mitochondrial fission and mitophagy, collectively disrupting homeostasis and antioxidant defenses. These findings point to mitochondrial dysfunction and impaired homeostasis in IBD mice, with reduced antioxidant capacity. Importantly, EA normalized these perturbations. It reduced pathological DRP1/PARK2 levels, restored FtMt expression, and mitigated ferroptotic ultrastructural damage. This demonstrates that EA stabilizes mitochondrial dynamics by modulating fission and mitophagy pathways, thereby preserving epithelial integrity in IBD through ferroptosis suppression.

In 2021, David Julius and Ardem Patapoutian were awarded the Nobel Prize in Physiology or Medicine for their discovery of the TRPV1 temperature receptor and the Piezo touch receptor. This brought Piezo to the forefront of academic and public attention once again. The discovery of Piezo proteins has uncovered the molecular mechanisms of mechanical force perception, offering an important new perspective on how cells sense mechanical signals. Mechanical forces are widespread in the digestive tract, and mechanically sensitive ion channels can rapidly convert mechanical signals into electrochemical signals, participating in many physiological processes related to mechanosensory transduction [13]. Intestinal epithelial cells are constantly subjected to external forces, such as shear stress from the movement of luminal contents and stretching and compression caused by peristalsis [12]. Alterations in the mechanical microenvironment can lead to pathological conditions such as infection, inflammatory diseases, and cancer [54]. When the gastrointestinal tract exhibits pathological changes such as dysmotility, inflammation, and luminal stenosis, these mechanical forces can adversely affect the biological behavior of intestinal epithelial cells, chromaffin cells, and other cells. Piezo, as a typical representative of mechanical force receptors, is expressed throughout the digestive system in Piezo1/2 forms, indicating its close relationship with the occurrence and development of digestive diseases [55]. Pharmacological studies have shown that the Piezo channel can be specifically blocked by mechanically sensitive channel blockers such as Gd^3+^ and ruthenium red, as well as by GsMTx4 [56]. Emerging research has revealed that enteric neuronal Piezo1 is predominantly expressed in cholinergic neurons, and silencing these neurons not only slows colonic motility but also limits aberrant intestinal inflammation [57]. Given accumulating evidence indicating that EA can engage distinct autonomic circuits, contingent upon acupoint selection or stimulation intensity, to modulate systemic inflammation [58, 59], an intriguing question is whether enteric neuronal Piezo1 participates in EA-activated neuromodulatory loops. Our study specifically focused on Piezo1 in the intestinal epithelium during IBD pathogenesis, as corroborated by parallel research demonstrating that genetic deletion or pharmacological inhibition of epithelial Piezo1 mitigates barrier damage by suppressing ferroptosis—identifying it as a therapeutic target in ulcerative colitis [60, 61]. Of note, prior studies have confirmed the broad expression of Piezo1 across multiple intestinal cell types. However, current evidence collectively suggests that Piezo1 in IECs and in the enteric nervous system exerts distinct regulatory roles in IBD-associated inflammation. We therefore propose a dual-mode mechanism in which EA initially activates enteric cholinergic neurons to release acetylcholine, which dampens local inflammation via α7nAChR-mediated cholinergic anti-inflammatory pathways. Concomitantly, EA downregulates mechanosensitive Piezo1 in the enteric nervous system, attenuating aberrant peristalsis and thereby reducing pathological mechanical stress on the epithelium. This mechanical relief suppresses epithelial Piezo1–Ca^2^⁺ signaling, preserves mitochondrial homeostasis, and ultimately prevents intestinal epithelial ferroptosis.

Piezo proteins, among the largest known plasma membrane ion channel complexes, directly sense mechanical forces and convert environmental mechanical signals into intracellular Ca^2^⁺ signals, initiating downstream responses [62]. Previous research has established Ca^2^⁺ as a key regulator of mitochondrial transport proteins, enzymes, and respiratory complexes [63]. In various diseases, increased Ca^2^⁺ influx is often accompanied by mitochondrial ROS accumulation [64]. Yan T et al. [65]discovered that elevated mtROS can activate JNK and p38 MAPK, increasing Drp1 phosphorylation and promoting mitochondrial fission and dysfunction. Additionally, intracellular Ca^2^⁺ accumulation activates CaMKII, which further phosphorylates Drp1, collectively disrupting mitochondrial membrane potential and function [66]. Recent studies have confirmed that Piezo1 can induce ferroptosis in chondrocytes and lung endothelial cells by disrupting mitochondrial homeostasis through calcium ion mediation [67, 68]. Furthermore, Piezo1 is highly expressed in the intestines of Crohn's disease patients. Activating Piezo1 in intestinal epithelial cells increases Ca^2^⁺ influx; Ca^2^⁺ overload leads to mitochondrial ROS accumulation and decreased membrane potential, causing mitochondrial damage and destabilizing mitochondrial homeostasis, thereby exacerbating intestinal inflammation [27]. Thus, abnormal mechanical forces in IBD can activate Piezo1, increasing Ca^2^⁺ influx, damaging mitochondrial homeostasis, disrupting antioxidant balance, causing lipid peroxidation, and inducing ferroptosis, further worsening IBD-related intestinal epithelial injury.

The selection of acupoints ST25 and ST36 was guided by both classical Traditional Chinese Medicine (TCM) principles and contemporary clinical evidence. According to meridian theory, ST25 and ST36 are indicated for resolving *damp-heat accumulation*—a core TCM pathogenesis of IBD. This reflects their established efficacy in regulating intestinal function through synergistic local-distal point combinations. From the perspective of modern acupuncture anatomy and neural pathways, ST25 is located at the T9 spinal segment; stimulation of this region can activate segmental somato-sympathetic reflexes to modulate intestinal function. ST36, innervated by the L4 spinal cord, can enhance vagus nerve tone when stimulated, thereby increasing the activity of the cholinergic anti-inflammatory pathway [69]. Furthermore, current evidence outlines a key neuro-regulatory circuit involving the vagus nerve-adrenal pathway associated with acupuncture at ST36, suggesting that acupuncture-driven activation of this pathway has beneficial anti-inflammatory effects [58]. Therefore, ST25 and ST36 were selected for this study based on their potential to alleviate both intestinal dysmotility and inflammation in IBD. However, stimulating different acupoints may exert distinct effects on IBD. For instance, Li et al. [70]found that electroacupuncture at the Dachangshu (BL25) acupoints for 7 days significantly alleviated visceral pain and depressive complications in IBD. Future studies should compare the specific disease-modifying mechanisms across different acupoint combinations to identify optimal therapeutic protocols.

As an adjuvant therapy for IBD, acupuncture has demonstrated promise in preclinical studies. Animal models of IBD have provided evidence that electroacupuncture can decrease disease activity and inflammation. For instance, electroacupuncture has been shown to reduce the production of pro-inflammatory cytokines such as TNF-α and IL-1β, while boosting the levels of anti-inflammatory cytokines like IL-10 [71, 72]. This immune modulation is partly mediated by the vagus nerve, which plays a crucial role in inflammation regulation through the cholinergic anti-inflammatory pathway. By stimulating the vagus nerve, electroacupuncture can inhibit the release of pro-inflammatory cytokines, thereby reducing intestinal inflammation [73, 74]. Moreover, acupuncture has been proven to regulate the gut microbiota, a key factor in IBD pathogenesis. Studies have indicated that the therapeutic effects of acupuncture are associated with an increased abundance of anti-inflammatory gut bacteria and SCFAs, enhanced intestinal barrier function, and inhibition of Th1/Th17-related pro-inflammatory cytokines [8]. This restoration of gut microbiota balance may aid in improving intestinal barrier function and reducing inflammation. Nevertheless, the clinical application of acupuncture in IBD is still in its early stages, with limited and diverse evidence from clinical trials. While some studies indicate that acupuncture may benefit symptoms such as abdominal pain, diarrhea, and quality of life in IBD patients [8, 18, 75], further well-designed, large-scale clinical trials are needed to confirm these findings and establish standardized acupuncture protocols for IBD treatment.

Previous studies have shown that the activation of the Piezo1 channel under mechanical forces can lead to ferroptosis in chondrocytes [68]. In the in vitro experiments of this study, the activation of the Piezo1 channel was also found to be associated with ferroptosis in IECs. Mechanical stimulation or treatment with Yoda1 potentiated Piezo1 channel opening, which triggered pathognomonic ferroptosis signatures, including GSH and GPX4 depletion, as well as elevated levels of ROS, MDA, Ca^2^⁺, Fe^2^⁺, and mitochondrial damage. When the Piezo1 channel was inhibited using GsMTx4, the ferroptosis changes and oxidative stress caused by mechanical stimulation were almost completely eliminated. Interestingly, the addition of the ferroptosis regulator Fer-1 could rescue ferroptosis and oxidative injury caused by mechanical stimulation, indicating that Piezo1 activated by mechanical stimulation does mediate ferroptosis in intestinal epithelium. These findings were mirrored in vivo, where Piezo1 upregulation correlated strongly with clinical severity markers, including elevated DAI, accelerated intestinal transit, and pro-inflammatory cytokines. After administering GsMTx4 to IBD mice, the function of Piezo1 was inhibited, with a downward trend in its expression, and the pathological damage to the colon was alleviated. Notably, GsMTx4 treatment restored the excessive mitochondrial fission and autophagy, increased the levels of ferroptosis-related proteins GPX4, FTH, and GSH, and decreased the levels of MDA and Fe^2^⁺. This confirmed that the activation of Piezo1 can lead to mitochondrial homeostasis imbalance and trigger ferroptosis in the intestinal epithelium of IBD mice.

Furthermore, similar to GsMTx4, EA mimicked the effects of Piezo1 inhibition by downregulating its expression and alleviating IBD pathology. Even under the Yoda1 challenge, EA partially countered Piezo1 hyperactivation by upregulating FtMt, GPX4, and FTH, while also suppressing DRP1, PARK2, MDA, Fe^2^⁺, and inflammatory cytokines. This coordinated response preserved mitochondrial homeostasis and antagonized ferroptosis. Collectively, these findings suggest EA may exert unique therapeutic advantages in regulating the Piezo1-mitochondria-ferroptosis axis. Piezo1 activity itself, together with the mitochondrial integrity markers DRP1 and PARK2 and the ferroptosis signature molecules GPX4 and FTH, could serve as biomarkers for EA treatment or IBD activity. Future studies should therefore incorporate these targets for diagnostic or therapeutic purposes and optimize EA protocols—by refining stimulation parameters and acupoint combinations—to modulate this axis more precisely and enhance clinical translation. In summary, EA may suppress ferroptosis in IBD intestinal epithelial cells by modulating Piezo1-mediated mitochondrial homeostasis.

This study has limitations as well. Although the DSS-induced acute colitis model efficiently recapitulates key inflammatory features of IBD, it does not fully model the chronic, relapsing pathophysiology of human IBD, which involves progressive fibrosis, immune cell reprogramming, and extraintestinal manifestations. This limitation precludes direct extrapolation of our findings to long-term disease management or complications such as intestinal stricturing. Additionally, clinical IBD is frequently comorbid with depression or anxiety via gut-brain axis dysregulation. Future research could develop a chronic IBD model with multiple time-point observations to explore the long-term effects of EA and the recurrent features of IBD.

## Conclusion

This study establishes ferroptosis as a critical pathomechanism in IBD, demonstrating that Piezo1-mediated mitochondrial dyshomeostasis drives intestinal epithelial injury. Through integrated in vitro and in vivo approaches, we validate that mechanical activation of Piezo1 disrupts mitochondrial integrity, precipitating ferroptosis—a cascade pharmacologically rescued by Piezo1 inhibition or ferroptosis suppression. Crucially, we identify EA as a regulator of the Piezo1-mitochondria-ferroptosis axis, preserving mitochondrial function and redox equilibrium to attenuate epithelial damage. These findings not only advance the pathophysiological framework of IBD but also propose novel therapeutic strategies targeting mechano-metabolic crosstalk in gut inflammation.

## Supplementary Information

Additional file 1** Supplementary Fig. 1** EA regulates mitochondrial homeostasis and oxidative stress via Piezo1 in IBD. **A** Western blot bands. **B**–**D** Western blot analysis of FtMt, DRP1, and PARK2 expression (n = 3). **E**–**G** Biochemical assays for MDA, GSH, and Fe^2^⁺ levels (n = 5). *p < 0.05, **p < 0.01, ***p < 0.001 vs PBS + EA group.

## Data Availability

No datasets were generated or analysed during the current study.

## Abbreviations

ACSL4: Acyl-CoA synthetase long-chain family member 4
CD: Crohn’s disease
CMDI: Colonic mucosal damage index
DAI: Disease activity index
DRP1: Dynamin-related protein 1
DSS: Dextran sulfate sodium
EA: Electroacupuncture
Fer-1: Ferrostatin-1
FTH: Ferritin
FtMt: Mitochondrial ferritin
GPX4: Glutathione peroxidase 4
GSEA: Gene Set Enrichment Analysis
GSH: Glutathione
HIEC-6: Human Intestinal Epithelial Cell-6
IBD: Inflammatory bowel disease
IECs: Intestinal epithelial cells
MDA: Malondialdehyde
MF: Mechanical force
PARK2: Parkinson protein 2
ROS: Reactive oxygen species
SCFAs: Short-chain fatty acids
SOD: Superoxide dismutase
TCM: Traditional Chinese Medicine
TEM: Transmission electron microscopy
UC: Ulcerative colitis
