# Supplementary material for: Electroacupuncture attenuates intestinal epithelial ferroptosis in inflammatory bowel disease via Piezo1-mediated mitochondrial homeostasis
Source: Chin Med. 2025 Oct 6;20:161. doi: 10.1186/s13020-025-01218-7 (PMC12498448; doi:10.1186/s13020-025-01218-7)
Supplement: Supplementary file 1 — Additional file 1 Supplementary Fig. 1 EA regulates mitochondrial homeostasis and oxidative stress via Piezo1 in IBD. A Western blot bands. B–D Western blot analysis of FtMt, DRP1, and PARK2 expression (n = 3). E–G Biochemical assays for MDA, GSH, and Fe2⁺ levels (n = 5). *p < 0.05, **p < 0.01, ***p < 0.001 vs PBS + EA group. [file 13020_2025_1218_MOESM1_ESM.docx]

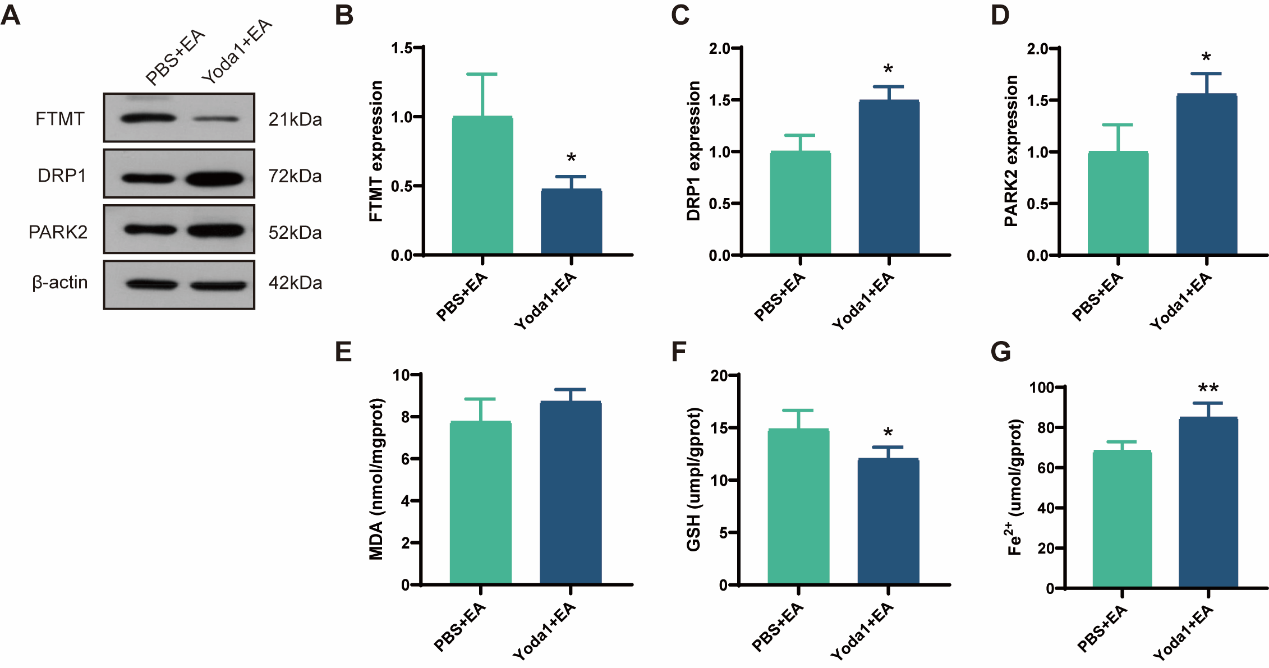


**Supplementary Fig. 1** EA regulates mitochondrial homeostasis and oxidative stress via Piezo1 in IBD. **A** Western blot bands. **B-D** Western blot analysis of FtMt, DRP1, and PARK2 expression (n=3). **E-G** Biochemical assays for MDA, GSH, and Fe²⁺ levels (n=5). * *p*<0.05, ** *p*<0.01, *** *p*<0.001 vs PBS+EA group.
